# Low Loss Nanostructured Polymers for Chip-scale Waveguide Amplifiers

**DOI:** 10.1038/s41598-017-03543-w

**Published:** 2017-06-13

**Authors:** George F. R. Chen, Xinyu Zhao, Yang Sun, Chaobin He, Mei Chee Tan, Dawn T. H. Tan

**Affiliations:** 1 Engineering Product Development, Singapore University of Technology and Design, 8 Somapah Road, Singapore, 487372 Singapore; 2 Department of Materials Science and Engineering, National University of Singapore, 9 Engineering Drive 1, Singapore, 117576 Singapore; 3 Institute of Materials Research and Engineering, Agency for Science, Technology and Research (A*STAR), 3 Research Link, Singapore, 117602 Singapore

## Abstract

On-chip waveguide amplifiers offer higher gain in small device sizes and better integration with photonic devices than the commonly available fiber amplifiers. However, on-chip amplifiers have yet to make its way into the mainstream due to the limited availability of materials with ideal light guiding and amplification properties. A low-loss nanostructured on-chip channel polymeric waveguide amplifier was designed, characterized, fabricated and its gain experimentally measured at telecommunication wavelength. The active polymeric waveguide core comprises of NaYF_4_:Yb,Er,Ce core-shell nanocrystals dispersed within a SU8 polymer, where the nanoparticle interfacial characteristics were tailored using hydrolyzed polyhedral oligomeric silsesquioxane-graft-poly(methyl methacrylate) to improve particle dispersion. Both the enhanced IR emission intensity from our nanocrystals using a tri-dopant scheme and the reduced scattering losses from our excellent particle dispersion at a high solid loading of 6.0 vol% contributed to the outstanding optical performance of our polymeric waveguide. We achieved one of the highest reported gain of 6.6 dB/cm using a relatively low coupled pump power of 80 mW. These polymeric waveguide amplifiers offer greater promise for integrated optical circuits due to their processability and integration advantages which will play a key role in the emerging areas of flexible communication and optoelectronic devices.

## Introduction

Amplification of optical fields is a fundamental function needed in the processing of optical signals. The amplification of light is performed via several processes, with rare-earth (RE) doped amplification being one of the most useful techniques. To date, erbium (Er) doped fiber amplifiers are responsible for the proliferation of data transmission since the ^4^I_13/2_ → ^4^I_15/2_ transition (~1530 nm) of Er ions falls within the low-loss telecommunication window of 1300–1650 nm^1–3^. In these amplifiers, an optical pump located at 980 nm enables amplification of a signal within the 1530–1600 nm region.

While Er-doped fiber amplifiers have achieved much success in long-haul telecommunication networks, chip-scale demonstrations of such amplifiers have remained less prolific. One of the issues associated with chip-scale demonstrations includes the difficulty in uniformly doping inorganic solid-state crystals or glasses with Er ions to create highly emissive active materials^4, 5^. Er ions are typically incorporated within ceramic or glass materials (e.g., SiO_2_) using costly physical methods such as melt processing, molecular beam epitaxy, ion implantation or laser deposition. The low solubility and non-uniform distribution of Er ions in conventional ceramics and glasses pose an additional barrier towards fabricating low-loss monolithic ceramic waveguides. One of the approaches to circumvent these limitations is to disperse active fillers of Er-doped inorganic nanocrystals within polymers to form polymeric-based waveguides as an alternative to inorganic RE doped ceramic amplifiers^6–10^. In this structure, Er ions are doped within inorganic nanocrystals which are dispersed in the polymer such that emission quenching that arises from Er ion interactions with organic functional groups (e.g., -OH, -CH) commonly found in polymers can be avoided. Moreover, the undesired concentration quenching effects (i.e. arising from clusters of high Er concentration) often experienced in ceramic glasses and amorphous organic-polymer systems can be limited using these nanoparticle-polymer waveguide nanocomposites. Furthermore, these polymeric waveguide amplifiers with its greater promise for integrated optical circuits due to their processability and integration advantages will also play a key role in the emerging area of flexible communication and optoelectronic devices.

To achieve highly emissive nanocomposites with low optical losses which is essential for constructing high gain polymeric-based amplifier, the following requirements need to be fulfilled: (1) bright infrared (IR)-emitting RE doped nanocrystals, (2) high nanocrystal loading and (3) uniform nanocrystal dispersion within the polymer matrix. The selection of suitable RE dopants (e.g. sensitizers and activators) are crucial towards the fabrication of RE-doped nanocrystals that absorb and emit at the desired wavelength. Furthermore, the concentration of RE dopants would have to be optimized. Whilst a low concentration of the emitting centers leads to weak IR emissions, high concentrations of RE emitting centers would also result in a reduction of IR emissions due to the concentration quenching effect^11^. Besides the dopant choices and concentration, the host chemistry and heterostructure are just as important towards obtaining high efficiency IR-emitting RE doped nanocrystals. In this work, hexagonal-phase NaYF_4_ nanocrystal was synthesized as the host for the RE dopants, since it is one of the most efficient IR-emitting host. It should also be noted that the hexagonal-phase NaYF_4_ emits up to 10 times brighter than its cubic-phase counterpart due to its low phonon energy which promotes radiative relaxation, and favorable lattice sites for substitution with lanthanide ions^11, 12^. High loading of these IR-emitting nanoparticles is another contributing factor that dictates the achievable gain of the polymeric waveguide. However, at high nanoparticle loading, nanoparticle agglomeration due to their large specific surface areas results in undesirable scattering losses. Subsequently, the nanoparticles’ surface properties should be tailored to improve their dispersion by inhibiting the agglomeration of these RE doped nanocrystals. We have recently successfully used a surfactant molecule prepared at our lab, hydrolyzed polyhedral oligomeric silsesquioxane-graft-poly(methyl methacrylate) (H-POSS-PMMA), to effectively avoid agglomeration of IR-emitting nanocrystals. Consequently, a mostly single particle dispersion at 10 vol% was observed leading to enhanced thermal stability and reduced scattering losses^13^.

In this paper, we aim to design and fabricate hexagonal-phase RE-doped NaYF_4_ nanocrystals that are dispersed within SU8 (an epoxy-based photoresist) to create a polymeric waveguide amplifier (PWA). The dopants selected for our IR-emitting NaYF_4_ nanocrystals have been optimized to maximize the IR emission intensities. In addition, we seek to achieve a uniform particle dispersion with high solid loading by modifying these brightly-emitting nanocrystals with H-POSS-PMMA to inhibit the extent of agglomeration. We will also discuss about the device architecture design used to maximize the extent of the optical field residing within the waveguide. The waveguide amplifier was fabricated in this work using a deep-dicing technique which also enables the measurement of gain for varying device lengths.

## Design and Results of Low-Loss Nanostructured PWA

### Synthesis of Bright IR-Emitting Nanoparticles

**Figure 1:**
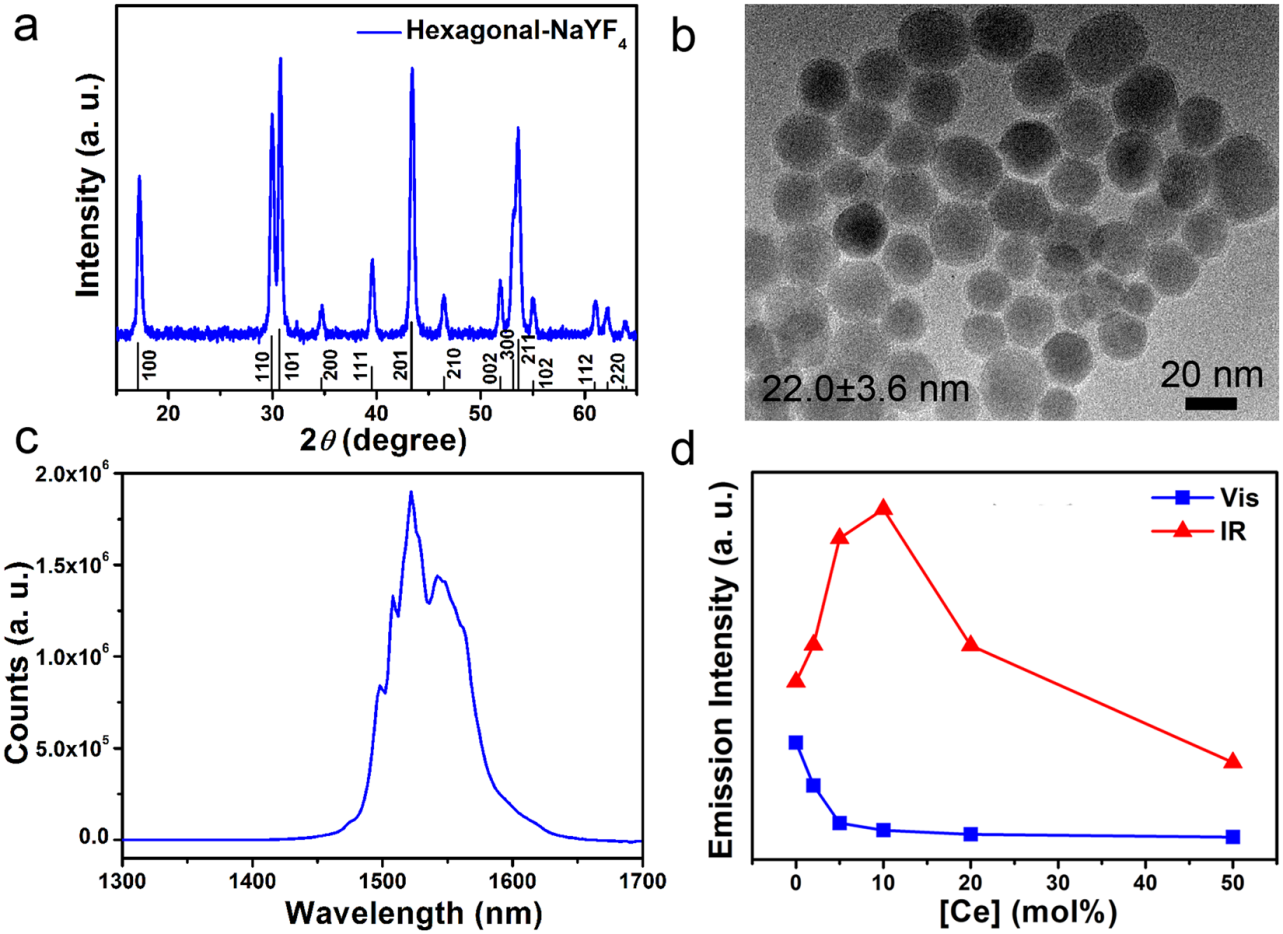
Characterization of our brightly IR-emitting NaYF_4_:Yb_20_Er_2_Ce_10_ core-shell nanoparticles (N-NPs). (**a**) XRD pattern showing hexagonal structure; (**b**) TEM image showing the N-NP size distribution; (**c**) Steady-state IR emission spectrum showing characteristic peak emission at 1530 nm corresponding to the ^4^I_13/2_ → ^4^I_15/2_ Er transition; (**d**) Integrated IR emission intensity at different Ce concentrations for 20 mol% Yb and 2 mol% Er, where the optimum was at ~10 mol% of Ce.

Hexagonal phase NaYF_4_:Yb,Er,Ce core-shell nanocrystals (N-NPs) with excellent IR emission upon excitation at 975 nm was synthesized using a thermal decomposition method^14^. The XRD pattern shows that N-NPs consist of pure hexagonal NaYF_4_ phase (JCPDS 16-0334) (Fig. 1a). Using the Scherrer equation, the grain size for the N-NPs was estimated to be ~22.0 ± 0.4 nm. TEM image shows that the N-NPs of 22.0 ± 3.6 nm with a spherical morphology was synthesized (Fig. 1b and Figure S1). Since both particle and grain sizes were approximately the same, it indicates that single crystals of N-NPs were synthesized. The measured steady-state emission peak at ~1530 nm is attributed to the characteristic ^4^I_13/2_ → ^4^I_15/2_ transition of Er (see Fig. 1c). The undoped shell of the core-shell N-NPs is critical as it effectively limits any quenching that typically arises from surface defects or quenching groups (e.g., OH and CH_2_ groups), and shields the Er emitting centers in the core. Furthermore, our tri-dopant scheme of Ce, Yb and Er has been shown to enhance the IR emission through a proposed phonon-assisted energy transfer processes^14–18^. By introducing Ce, the undesired visible emission of Er that accompanies its IR emission is quenched, while the IR emission intensity is enhanced through a proposed phonon-assisted energy transfer pathway^14^. The integrated IR emission intensity of our Ce,Yb,Er-doped N-NPs is ~2.4 times of that of NaYF_4_:Yb,Er nanocrystals (see Fig. 1d and Figure S2). Subsequently, the optimized Ce dopant concentration of 10 mol% was used in this work (see Fig. 1d). The estimated decay time of the N-NPs was estimated to be ~5.85 ms for the ~1530 nm transition by fitting the decay curve using a single exponential equation of *I* = *I*_0_exp(− *t*/*τ*), where *I*_0_ is the initial emission intensity and *τ* is the fitted decay time (see Figure S2c). Our reported decay time is slightly higher than the reported decay time of ~5.32 ms for NaYF_4_:Yb,Er core-shell nanocrystals with the similar size^19^. The longer decay time of our tri-doped nanocrystals indicates brighter IR emissions and higher efficiencies than conventional NaYF_4_:Yb,Er core-shell nanocrystals, which is also consistent with earlier observations^14^.

### Design of Waveguide Structure to Reduce Bending Losses

**Figure 2:**
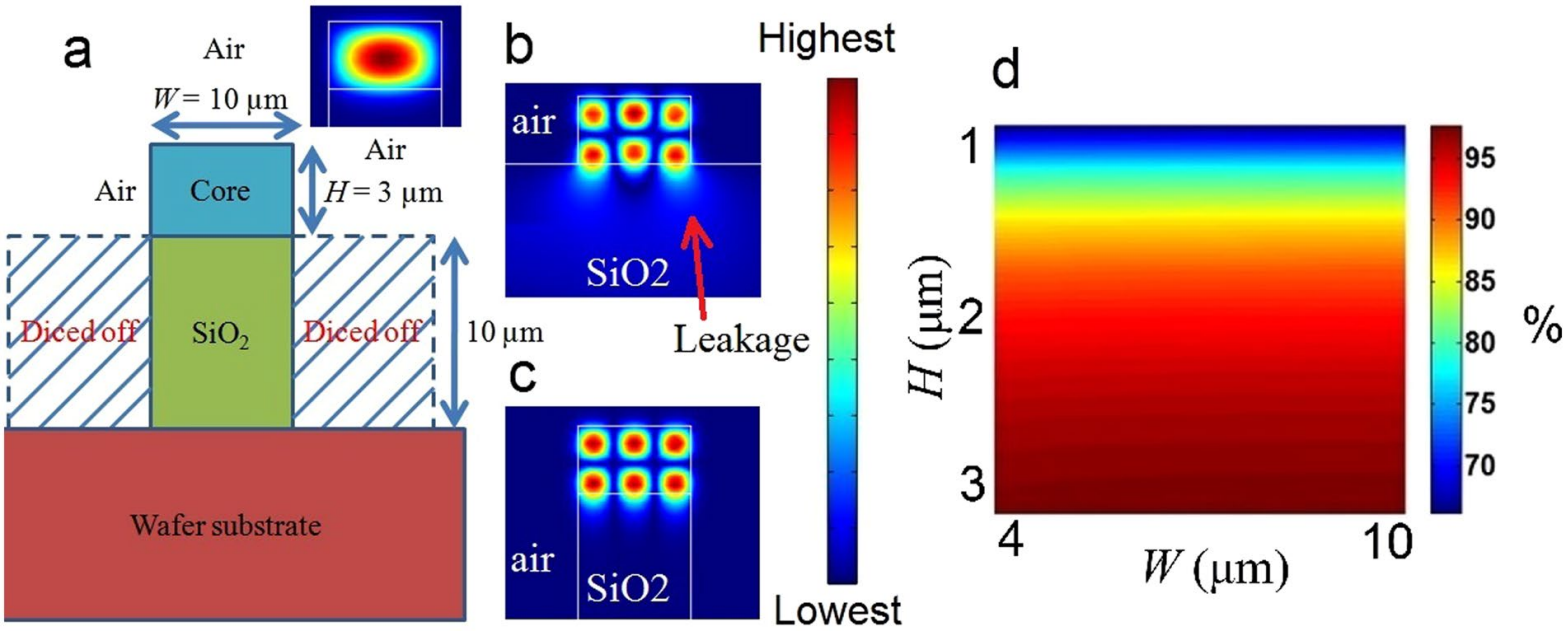
Design of proposed high gain air cladded channel waveguide and the theoretical modeling of its performance. (**a**) Cross section design of PWA; simulated fundamental mode in subset (**b**) leaky higher order mode, and (**c**) non-leaky higher order mode of our designed PWA (**d**) Optical field confinement of our PWA.

The refractive index of most optical polymers falls within the range of 1.49–1.58^10^, which is very similar to that of our N-NPs (i.e., 1.55)^6, 20, 21^. The small index mismatch between the N-NP and the polymer matrix (i.e., Δn = 0.03 to 0.06) leads to reduced scattering losses within the active PWA core during light propagation. However, the refractive index range of our PWA is also relatively close to that of the SiO_2_ cladding (i.e., 1.46), leading to low index contrast (i.e., Δn = 0.03 to 0.12). The low index contrast would consequently result in large bending losses within the device^22^. Therefore, an alternative design of the waveguide structure for such low index contrast PWA systems is warranted. Although the rib waveguide is a popular configuration in a low index contrast environment, its design would lead to high bending losses. It can be engineered to a single mode waveguide amplifier^7^ eliminating intermodal dispersion. In our proposed waveguide design, we would expose the waveguide’s core to air on three sides (see Fig. 2a), forming an air-cladded channel waveguide in order to maximize the modal confinement. Since the refractive index of air is 1, our index contrast up can be increase by up to ~0.58. Therefore, our air-cladded configuration increases the index contrast, which in turn increases light confinement in our waveguide leading to higher modal intensity. In addition, compared to the rib waveguide configuration which requires a horizontal layer above the core and leads to much longer device widths, our channel waveguide design would not require additional layers (i.e. smaller device footprint). In summary, a potential benefit of these two enhancements is lower bending loss, if a bend or a ring is required in the footprint.

In this work, we adopted the diamond blade dicing method to create straight air grooves to form rectangular channel waveguides (Fig. 2a)^23, 24^. This proposed fabrication method is cost-effective, simple, and does not require lithography and chemical etching. The downside of our selected fabrication method is some of the higher order modes could be leaky modes (Fig. 2b). We have addressed this shortcoming by dicing off more of the SiO_2_ sides (i.e., increasing the shaded portion in Fig. 2a), which eliminates the leaky nature (Fig. 2c). Higher mode confinement within the core means that a higher percentage of photons would interact with the N-NPs, thus increasing gain. Using n_SU8_ = 1.58, we used the beam propagation method in COMSOL software to calculate the mode field distribution (MFD) of the waveguide at 1530 nm. The percentage of mode field confined for the fundamental mode within the core for different widths (W, 4 to 10 μm) and heights (H, 1 to 3 μm) is also modeled and calculated (Fig. 2d). It is observed that the percentage confinement is relatively insensitive to the variation of the waveguide width but increases much more with waveguide height. For H = 3 μm, the percentage of power confined in the core is ~98.2% for the fundamental mode. This represents an improvement to rib waveguides with power confinement in the range of 80~91%. We choose H = 3 μm to have an almost complete optical confinement of the fundamental mode as the confinement improvement is insignificant when H is >3 μm. We select W = 10 μm since there is negligible differences in optical confinement of the fundamental mode as width varies. These choices maximize the optical field residing within the core.

### Fabrication of Low-Loss Nanostructured PWA

**Figure 3:**
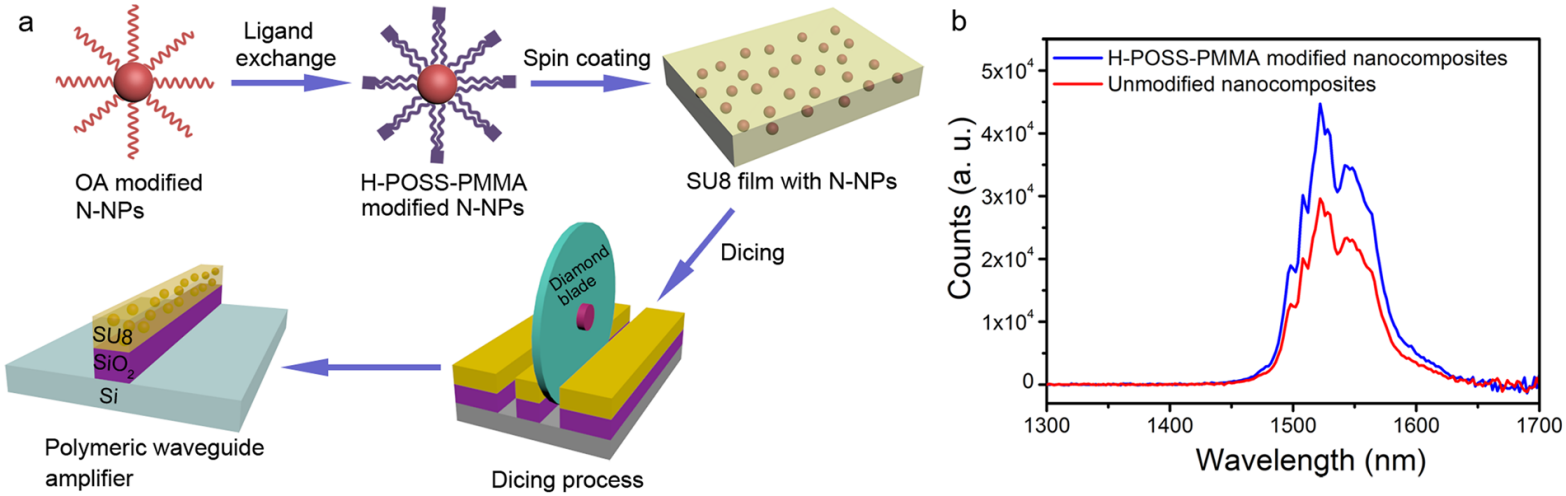
Fabrication and IR emissive properties of our active polymeric waveguide core. (**a**) Design and workflow for the fabrication of our polymeric waveguide amplifier. N-NPs represents NaYF_4_:Yb,Er,Ce core-shell nanoparticles; (**b**) IR emission spectra of polymer nanocomposites doped with H-POSS-PMMA modified and unmodified N-NPs. The enhanced IR emission of H-POSS-PMMA modified N-NPs nanocomposites is attributed to the decreased scattering losses due to excellent particle dispersion.

**Figure 4:**
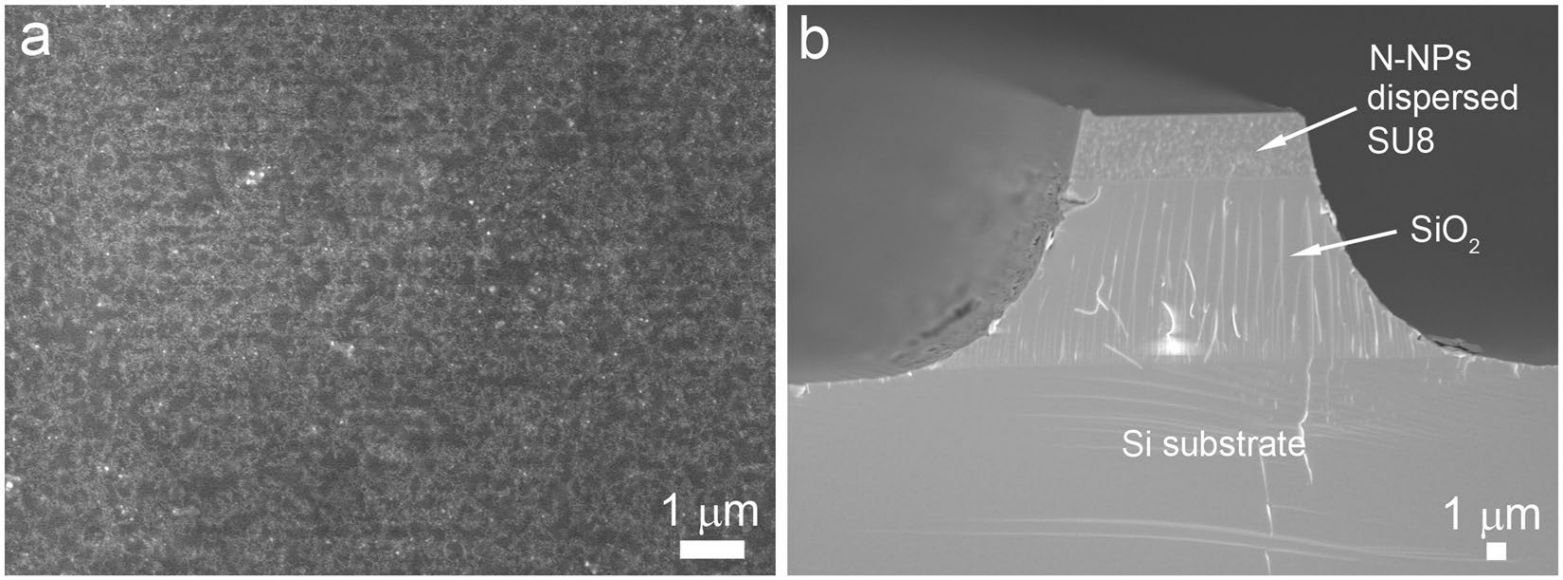
Microstructure of uniformly dispersed N-NPs in SU8 at a high loading of 6 vol%. SEM showing the (**a**) top view of N-NPs dispersed SU8 nanocomposite and (**b**) cross section of the fabricated polymeric waveguide amplifier on the SiO_2_ layer on a Si substrate.

Figure 3a shows a schematic of the design and workflow for the fabrication of low-loss nanostructured on-chip air-cladded channel PWA. We have used a ligand exchange method to modify the surface of our as-synthesized N-NPs with an amphiphilic polymer of H-POSS-PMMA to stabilize the N-NPs dispersion in solution and inhibit any aggregation in the polymer matrix. The H-POSS-PMMA replaces the original oleic acid surfactants on N-NP surfaces due to its higher carboxyl density which facilitates the ligand exchange process^13^. After ligand exchange, the H-POSS-PMMA modified N-NPs were dispersed in the SU8 solution (i.e. a solution of an epoxy-based photoresist), which was then spin-coated to form the N-NPs dispersed SU8 nanocomposite film. The enhanced IR emission of H-POSS-PMMA modified nanocomposites is attributed to the decreased scattering losses due to reduced agglomerate sizes and improved particle dispersion (Fig. 3b). The particle loading of N-NPs in the SU8 was estimated to be ~6.0 vol% (or 18.3 wt%, see Figure S3) using thermogravimetric analysis method. When we compared the percentage loading achieved here with that from most reported articles where N-NP loading is <<3 vol%, our loading is at least twice as much whilst maintaining a uniform dispersion (Fig. 4a)^11^. Then the N-NPs dispersed SU8 nanocomposite film on the SiO_2_ substrate was diced using a diamond blade to obtain an air-cladded channel waveguide in order to maximize the modal confinement as designed in Fig. 2a. The cross-section of our PWA was characterized using SEM (Fig. 4b), which shows that the width of our PWA is ~10.7 μm and the height is ~3.0 μm. These obtained dimensions of our PWA conform well to our designed parameters (Fig. 2), which confines ~98.2% for the fundamental mode according to our beam propagation simulation (Fig. 2d). The high N-NP loading achieved with uniform dispersion will be beneficial towards a more intense emission upon excitation and thus offer potential for higher gain from our active PWA core (Fig. 4a).

### Gain Measurements of our PWA

**Figure 5:**
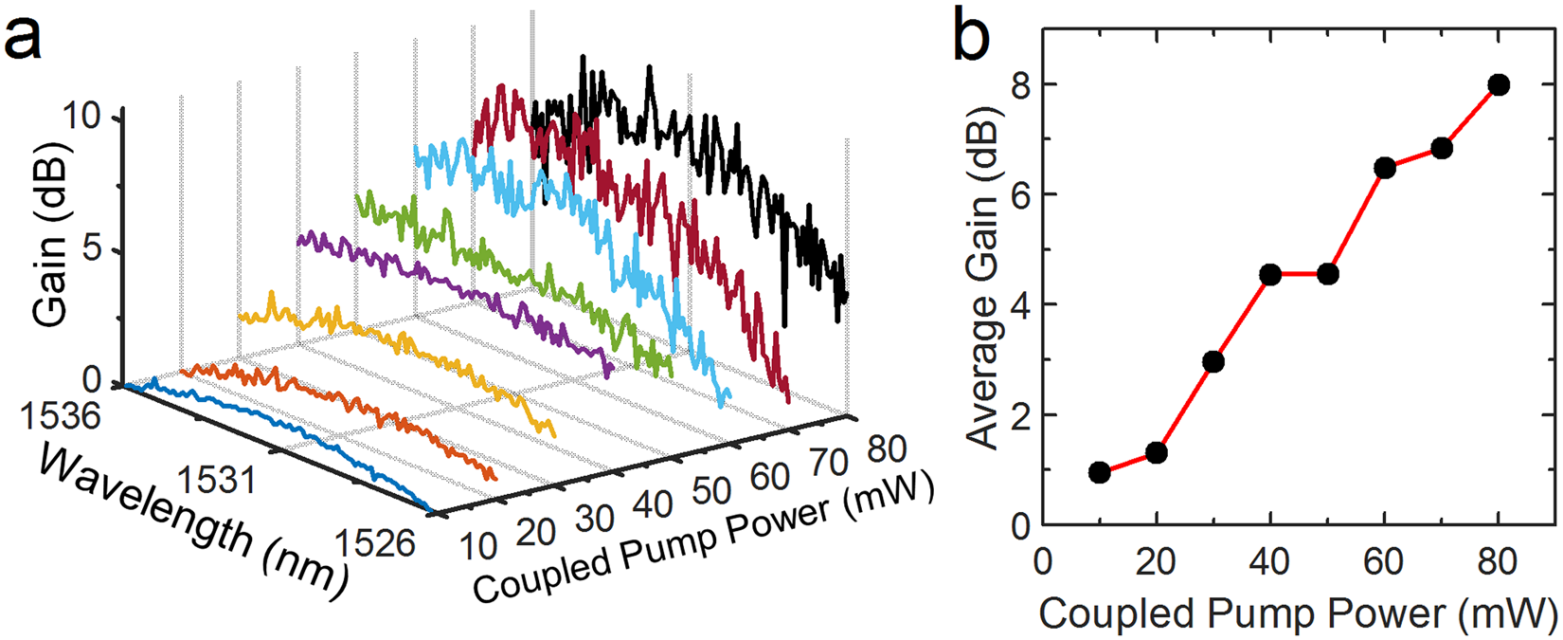
Measured gain performance from our air cladded PWA structures. (**a**) Relationship of gain with respect to wavelength and coupled pump power, and (**b**) Relationship of average gain with respect to coupled pump power.

The detailed experimental procedure used to measure the gain from our air cladded PWA is described in the Methods section. Using the cutback method, we measured a propagation loss of ~2.6 dB/cm and ~2.9 dB/cm for pure SU8 (i.e. no nanoparticles) and our SU8 nanocomposites containing the tri-doped N-NPs, respectively. Therefore, the addition of the uniformly dispersed tri-doped N-NPs contributed a negligible ~0.3 dB/cm of additional loss. The measured fiber-waveguide interface coupling loss was ~3 dB per facet. Considering that the pump power was varied from 20 to 160 mW and the fiber-waveguide interface has a loss of ~3 dB, the actual coupled pump power into the waveguide is 10 to 80 mW for an input signal power that was set to 0.1 mW. The gain was measured by normalizing the power obtained (using the optical spectrum analyzer, OSA) with the power of the amplified spontaneous emission (ASE) source. The relationship for the measured gain performance and average gain with respect to the couple power is shown in Fig. 5. We observed that the peaks of the gain measurements are within wavelength between 1530 to 1533 nm which is consistent with our earlier results from Figs 1 and 3.

From the results shown in Fig. 5, the maximum average gain achieved was 8 dB at coupled pump power of 80 mW. The maximum average gain achieved here was measured at relatively lower coupled pump power required and is ideal for applications for requiring low power consumption. Since the length of the waveguide is 1.2 cm, this would in turn represents a per unit device gain of 6.6 dB/cm. Compared with earlier reports of ~1.77 to 1.87 dB/cm using an identical material system (i.e. NaYF_4_ nanocrystal hosts in various polymers) at comparable coupled pump powers of 80 mW^16, 25^, our reported unit length gain is ~3.5 times higher. Using alternative hosts such as BaYF_5_ which has the potential for having both bright visible and IR emissions, the reported unit length gain was in the range of ~2.72 to 3.46 dB/cm^9, 26^. Comparing with this BaYF_5_ system, our reported gain of 6.6 dB/cm was still ~2 times higher. Furthermore, our reported gain was still ~1.5 times higher than that of the most recent report of ~4.5 dB/cm at 80 mW (relative gain of ~13 dB for 1.3 cm, with insertion losses of 3.6 dB per facet, neglecting propagation losses) for a doped NaYF_4_ systems coated with an undoped NaLuF_4_ shell^7^. Among other concerns, Lu is also one of the most expensive elements due to its limited supply amongst the RE elements. The high gain achieved using our PWA system is attributed to 3 reasons: (1) the high IR emission efficiency from the N-NPs, (2) high N-NP loading of 6.0 vol% with a uniform dispersion in our active composite waveguide core; and (3) improved mode confinement design using air as cladding and by etching off the SiO_2_ sides.

## Conclusions

In summary, we have designed, fabricated and characterized the a polymeric-based waveguide amplifier, comprising of H-POSS-PMMA modified N-NPs dispersed within a SU8 polymer, that achieved a maximum device gain of 6.6 dB/cm at a relatively low coupled pump power of 80 mW. Our reported gain is amongst one of the highest measured gain for PWA system that is reported to date. In our design, the IR emission intensity of the N-NPs was enhanced 2.37 times using a tri-dopant scheme of Yb, Er and Ce compared to the existing highest IR-emitting NaYF_4_ hexagonal phase core shell nanoparticles. The amphiphilic polymer H-POSS-PMMA stabilizes the N-NPs dispersion in SU8 and inhibits any aggregation in the polymer matrix, leading to reduced scattering losses and higher gain output. Therefore, the high gain of our device is attributed to the enhanced IR emission from our tri-doped N-NPs and the high N-NPs loading of 6.0 vol% that is also uniformly dispersed in SU8. The maximum gain of 8 dB achieved at a relatively low corresponding coupled pump power of 80 mW is also beneficial for decreasing the total power consumption of the waveguide amplifier. These polymeric optical waveguide amplifiers with its greater promise for integrated optical circuits due to their processability and integration advantages will play a key role in the emerging area of flexible communication and optoelectronic devices.

## Methods

### Synthesis of Tri-doped N-NPs

All chemicals were purchased from Sigma-Aldrich (Sigma Aldrich, St. Louis, MO) and used as received without any further purification. The NaYF_4_:Yb,Er,Ce core-shell nanoparticles were synthesized by using a solvothermal decomposition method as reported elsewhere^14^. Briefly, a mixture of (CF_3_COO)_3_Y (0.68 mmol), (CF_3_COO)_3_Yb (0.20 mmol), (CF_3_COO)_3_Er (0.02 mmol), (CF_3_COO)_3_Ce (0.10 mmol), and CF_3_COONa (1.5 mmol) was dissolved in an organic solution containing 1-octadecene (2.0 mL), oleic acid (4.4 mL), and oleylamine (3.5 mL) in a 100 mL three-neck flask at 120 °C under argon gas flow. The obtained solution was next heated to 330 °C and kept at this temperature for 1 h in an argon environment under vigorous stirring. Next, a shell precursor solution containing (CF_3_COO)_3_Y (2.0 mmol), CF_3_COONa (1.5 mmol), oleylamine (2.0 mL) and oleic acid (3.0 mL) was added to enable the formation of core-shell nanoparticles. The reaction mixture was subsequently cooled down to room temperature and the products were precipitated with the addition of ethanol. The synthesized N-NPs, were separated by centrifugation, washed with hexane and ethanol for once, and re-dispersed in chloroform.

### Preparation of Low-Loss Active Nanocomposite Comprising of N-NPs Dispersed in SU8

The spin coating solution was prepared using a ligand exchange method using H-POSS-PMMA, where H-POSS-PMMA was synthesized using a method reported elsewhere^13^. In a typical experiment, H-POSS-PMMA (16 mg) was added into a chloroform solution (1.5 mL) containing tri-doped N-NPs (21 mg). Then, the solution was sonicated and then stirred overnight allowing the ligand exchange between H-POSS-PMMA and the original oleic acid on the N-NPs’ surface. The solution was completely evaporated to obtain H-POSS-PMMA modified N-NPs powders that were dispersed into chloroform (800 μL) solution containing SU8 3005 (100 μL, MicroChem Corp. Westborough, MA USA) by sonicating. Next, the as-obtained solution was spin-coated onto SiO_2_ layer (10 μm in thickness) on a Si substrate, using 6000 rpm for 1 min to form the H-POSS-PMMA modified N-NPs containing SU8 nanocomposite film. The nanocomposite film was baked at 65 °C for 3 min followed by 95 °C for 3 min to evaporate the solvent and solidify the SU8 polymer film before further fabrication and characterization.

### Fabrication of PWA Channel Structure

After coating our polymer waveguide core on the SiO_2_ layer on a Si substrate, we used commercial diamond blade dicing to deep-dice air slits to create air-cladded channel waveguide structures according to our selected design parameters. The blade used was resin-type 50 mm diameter, where grit and bonding strength were optimized. A rotation speed at 20,000 rpm was used for the dicing together with a feed speed of 0.1 mm/s. We set up the sample onto the sticky tape (120 μm) bounded by a metal rim frame, ensuring no air bubbles between the layers.

### Characterization of N-NPs

XRD patterns were measured on a D8 Eco Advance powder diffractometer (Bruker AXSInc., Madison, WI) using Cu Kα radiation with wavelength of 1.5418 Å (step size 0.02°, duration 0.5 s, working voltage 40 kV, working current 25 mA). Particle morphology of N-NPs was measured using a JEOL 2010 transmission electron microscope (JSM-7600F, JEOL Ltd., Japan) operating at an acceleration voltage of 200 kV. Steady state luminescence spectra were measured upon excitation with a 975 nm continuous wave laser (CNI MDL-III-975, Changchun New Industries Optoelectronics Tech. Co. Ltd., China) using a FLS980 Fluorescence Spectrometer (Edinburgh Instruments Ltd., UK) (laser power 20 mW, spot size of 19.63 mm^2^, step size 2 nm, dwell time 0.2 s). The powder samples were packed in demountable Spectrosil far UV quartz Type 20 cells (StarnaCells, Inc., Atascadero, CA) with 0.5 mm path lengths for emission collection. To measure the time-resolved luminescence spectrum, the excitation source was modulated using an electronic pulse modulator to obtain excitation pulse at pulse duration of 10 μs with a repetition rate of 10 Hz.

### Characterization of Low-Loss Active Nanocomposite Comprising of N-NPs Dispersed in SU8

Thermogravimetric analysis (TGA) was performed using the TGA Q50 analyzer (TA Instruments, USA) in a N_2_ environment at a heating rate of 10 °C min^−1^ from 30 to 800 °C. Electronic micrographs of the composite film and waveguide on the silicon substrate were taken on a field emission scanning electron microscopy (JSM-7600F, JEOL Ltd., Japan).

### Gain Measurements of our Air-Cladded PWA

**Figure 6:**
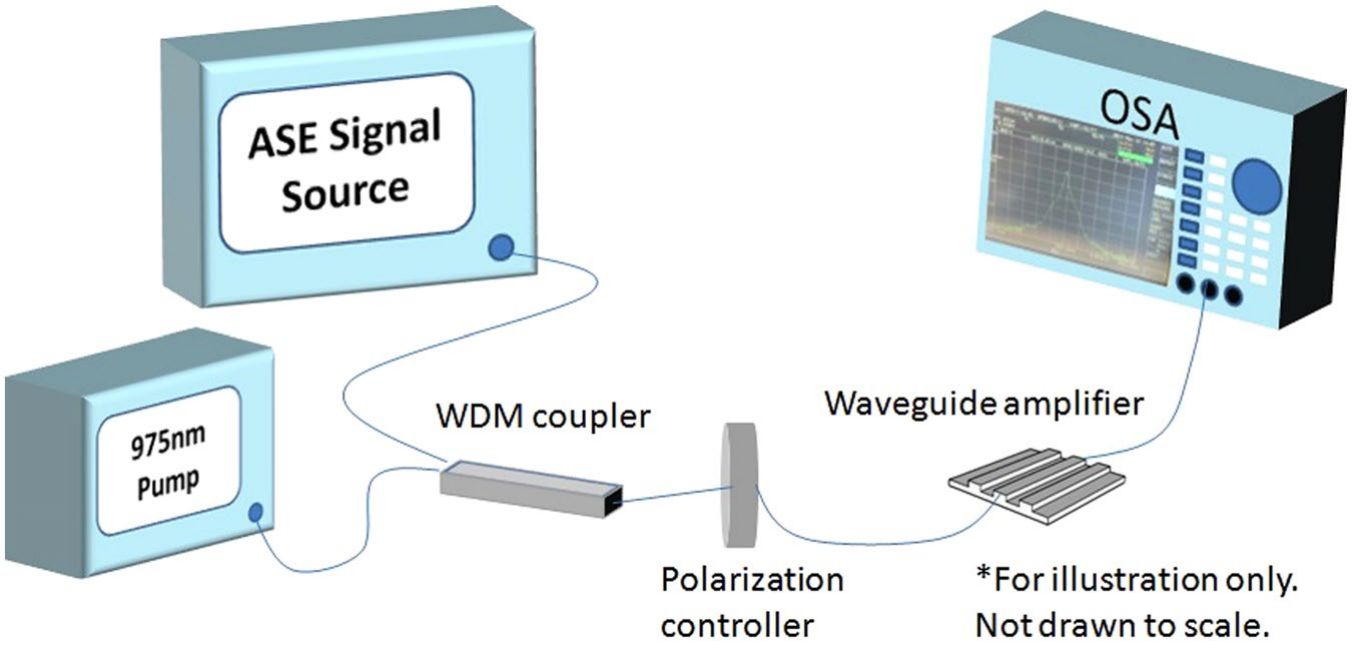
Waveguide device characterization setup used to measure the gain performance from our air-cladded PWAs with varying coupled pump powers.

We utilize an amplified spontaneous emission (ASE) source covering 1530–1600 nm that is connected to a fiber polarizer (see Fig. 6). Prior to experimentation, it was adjusted for the quasi-TE mode. The signal is combined with a 980 nm laser diode source as the pump source using a Wavelength Division Multiplexer fiber and coupled into the PWA core using a fiber as shown in Fig. 6. Alignment was maximized and the spectrum was recorded onto the Optical Spectrum Analyzer (OSA).

## Electronic supplementary material

Supplementary Information
