# Supplementary material for: Low Loss Nanostructured Polymers for Chip-scale Waveguide Amplifiers
Source: Sci Rep. 2017 Jun 13;7:3366. doi: 10.1038/s41598-017-03543-w (PMC5469753; doi:10.1038/s41598-017-03543-w)
Supplement: Supplementary file 1 — Supplementary Information [file 41598_2017_3543_MOESM1_ESM.doc]

Supporting information

**Low Loss Nanostructured Polymers for Chip-scale Waveguide Amplifiers**

George F. R. Chen^1,a^, Xinyu Zhao^1,a^, Yang Sun^2^, Chaobin He^2, 3^, Mei Chee Tan^1,*^ and
Dawn T. H. Tan^1,+^

1. Engineering Product Development, Singapore University of Technology and Design, 8 Somapah Road, Singapore 487372, Singapore.

2. Department of Materials Science and Engineering, National University of Singapore, 9 Engineering Drive 1, Singapore 117576, Singapore

3. Institute of Materials Research and Engineering, Agency for Science, Technology and Research (A*STAR), 3 Research Link , Singapore 117602 , Singapore

^a^ These authors contributed equally to this work

To whom correspondence should be addressed:

*[meichee.tan@sutd.edu.sg](mailto:meichee.tan@sutd.edu.sg) and ^+^[dawn_tan@sutd.edu.sg](mailto:dawn_tan@sutd.edu.sg)


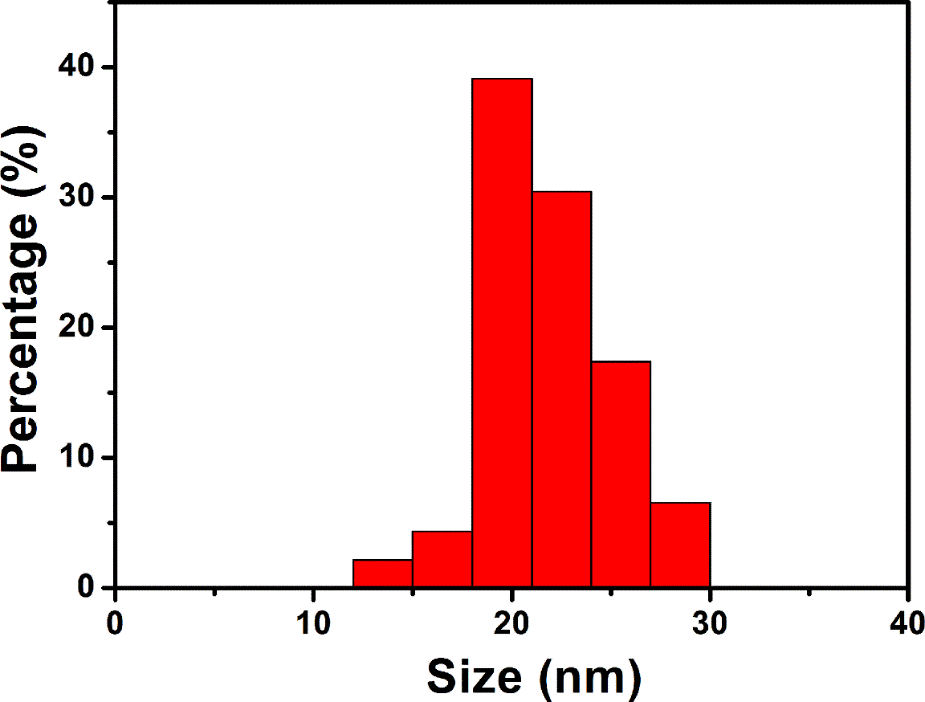


Figure S1. The TEM size distribution of NaYF_4_:Yb,Er,Ce core-shell nanocrystals (N-NPs). The average size of N-NPs is 22.0±3.6 nm.


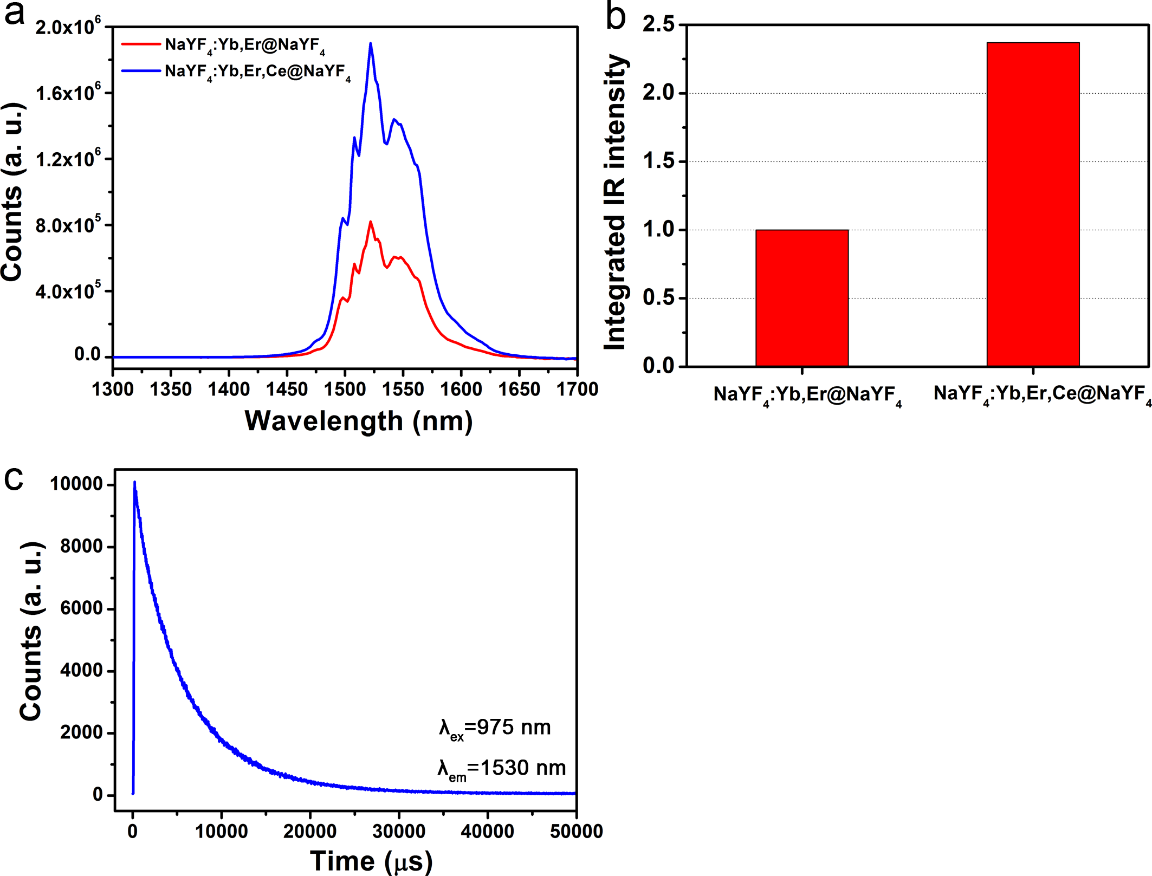


Figure S2. IR emission enhancement using our unique Yb, Er, Ce tri-dopant scheme. (a) IR emission spectra and (b) integrated IR emission intensity, of NaYF_4_:Yb,Er and NaYF_4_:Yb,Er,Ce core-shell nanocrystals; and (c) time-resolved luminescence spectrum of our NaYF_4_:Yb,Er,Ce core-shell nanocrystals (~5.85 ms).


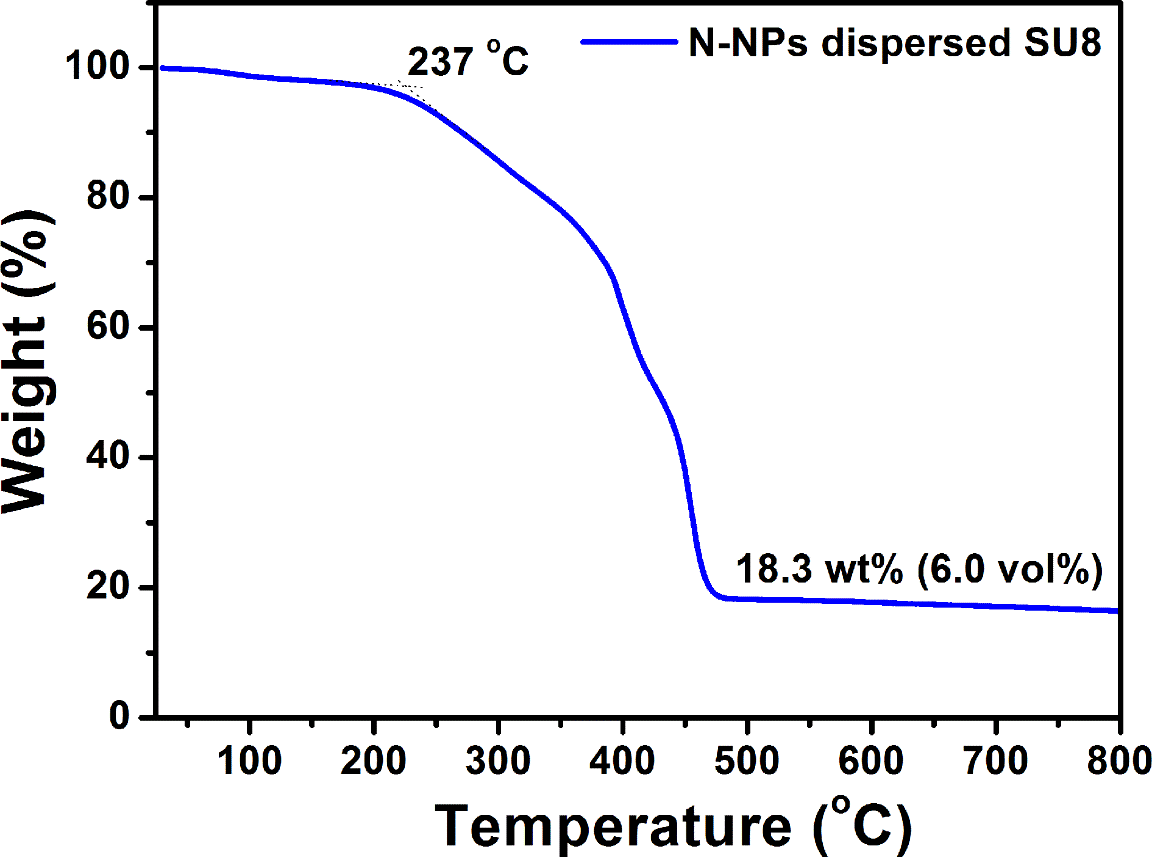


Figure S3. Thermal stability of our IR-active nanostructured polymeric waveguide from the TGA curve of NaYF_4_:Yb,Er,Ce core-shell nanocrystals dispersed SU8 nanocomposite film.
